# Multi–Output Classification Based on Convolutional Neural Network Model for Untrained Compound Fault Diagnosis of Rotor Systems with Non–Contact Sensors

**DOI:** 10.3390/s23063153

**Published:** 2023-03-15

**Authors:** Taehwan Son, Dongwoo Hong, Byeongil Kim

**Affiliations:** 1 Department of Mechanical Engineering, Korea Advanced Institute of Science and Technology, Daejeon 34141, Republic of Korea; 2 School of Mechanical Engineering, Yeungnam University, Gyeongsan 38541, Republic of Korea

**Keywords:** convolutional neural network, multi–output classification, non–contact sensors, rotor fault diagnosis, short–time Fourier transform

## Abstract

Fault diagnosis is important in rotor systems because severe damage can occur during the operation of systems under harsh conditions. The advancements in machine learning and deep learning have led to enhanced performance of classification. Two important elements of fault diagnosis using machine learning are data preprocessing and model structure. Multi–class classification is used to classify faults into different single types, whereas multi–label classification classifies faults into compound types. It is valuable to focus on the capability of detecting compound faults because multiple faults can exist simultaneously. Diagnosis of untrained compound faults is also a merit. In this study, input data were first preprocessed with short–time Fourier transform. Then, a model was built for classification of the state of the system based on multi–output classification. Finally, the proposed model was evaluated based on its performance and robustness for classification of compound faults. This study proposes an effective model based on multi–output classification, which can be trained using only single fault data for the classification of compound faults and confirms the robustness of the model to changes in unbalance.

## 1. Introduction

Rotor systems are widely used in industries for applications, such as motor systems, rotating shafts in automobiles, and hydroelectric generators [1,2]. It is important to detect faults in rotor systems because they affect safety, operation quality, and machine lifespan [3,4] (Industry 4.0 especially stresses prediction). Diverse types of faults can occur in rotor systems, such as misalignment, unbalance, looseness, shaft faults, and broken rotor bars [5]. The prediction of faults saves maintenance costs, which prolongs the operating life of a machine by preventing severe failures. Additionally, Industry 4.0 highlights prediction under factory conditions from the perspective of products [6]. Artificial intelligence (AI) has been extensively used in the diagnosis field because it outperforms conventional methods in prediction [7]. Recently, classification using machine learning (ML) and deep learning (DL) has gained significant attention for rotor fault diagnosis (RFD). One widely used ML method is the support vector machine (SVM) [8,9], and a widely used DL method is convolutional neural network (CNN) [10,11,12]. There are three main classification methods: multi–class, multi–label, and multi-output classification. Multi–class classification is used to classify each type of fault, whereas the remaining two methods are used for the classification of compound faults.

The selection of an appropriate signal processing method is as crucial as that of the classification method. Several types of signal processing method have been proposed, which include one–dimensional data (such as raw data) and two–dimensional data, including orbit, omnidirectional regeneration (ODR) [13], symmetrized dot pattern (SDP) [14,15], fast–Fourier transform (FFT), short–time Fourier transform (STFT) [11], continuous wavelet transform (CWT) [16], discrete wavelet transform (DWT) [14], Hilbert–Huang transform (HHT) [17], and synchrosqueezing transforms [18]. The last four transforms are time–frequency spectrograms, and this type of data is notably used as input data during classification. Recently, there has been work to enhance the impulsive signature in signals from rotating machinery, such as the kurtogram [19] and fast nonlinear blind decomposition [20].

In this study, STFT was used as the pre–processing method for the raw data acquired from a rotor kit. Subsequently, multi–output classification was performed using the proposed model and its robustness was confirmed using the input data extracted under ambiguous faults. The displacements were obtained from the eddy–current proximities in two perpendicular directions, X and Y, and were used as input data. Fifteen types of fault were analyzed (including four single fault types) for the training and test.

The remainder of this study is organized as follows: Section 2 discusses related works, Section 3 explains the proposed method, Section 4 presents the result, and Section 5 is a discussion of the results obtained.

The main contributions of this study are as follows.

1. A multi–output classification model was proposed to classify compound faults. Multi–output classification is an effective method when there are many types of fault. We diagnosed fifteen categories of fault including compound faults, but only four categories of sing fault were used to train the model. This is the big difference from the multi–class classification models, which are adopted for fault diagnoses widely. If we train a multi–class classification model, we need to categorize it into a large number of types, including compound faults. Then, the proposed model was compared with other conventional models to confirm its robustness.
2. Ambiguous fault data were applied to the model to confirm the robustness of the proposed model, and the independence of each probability with changes was analyzed. The probabilities in the results show that the proposed model classifies faults having weaker severity than the faults used to train.

## 2. Related Works

### 2.1. Input Data

Various data formats can be utilized as input data for ML and DL. First, the raw data are directly acquired from proximities and used as input data [9,10,21]. The orbit depicts two–dimensional data, while it does not contain time information. Kim et al. used ODR as the input data to improve the robustness of orbit [13]. Moreover, Zhu et al. and X. Zhu et al. selected SDP as input data, which was the transformed data of one–dimensional signal [14,15]. However, orbit, ODR, and SDP cannot express frequential information, whereas the following signal processing methods hold the information. FFT is a faster transforming method of discrete Fourier transform (DFT) and it provides frequency–amplitude information. However, FFT does not provide time information; thus, it is inappropriate for signals varying significantly with time. STFT applies Fourier transform time–locally to complement this disadvantage. Hence, it is a sequence of Fourier transforms of a windowed signal. Pham et al. proposed a model for multi-output classification of compound bearing faults with STFT images [11]. In addition, Rodriguez et al. used STFT for motor current signature analysis [22]. Although STFT can express time– and frequency–based information, it has a trade–off between the time and frequency resolutions depending upon the window size. The wavelet transform can be a solution to this problem because it resolves the trade–off between time– and frequency–resolutions by using windows of different sizes (a wide window for low–frequency regions and narrow window for high–frequency regions). Multiple studies have used CWT, DWT, and wavelet–based synchrosqueezing transform (WSST) as the input data to extract features of the signals [14,16,18]. Hilbert–Huang transform (HHT) and variational mode decomposition (VMD), which decompose signals into intrinsic mode functions (IMF), are effective signal analysis methods [17,23,24]. Wavelet transform (WT) and VMD are efficient methods to analyze signals changing locally, and HHT is a good method to describe instantaneous frequency signals. Research for impulsive signature enhancement and extraction is also conducted recently. Zongzhen Zhang et al. adopted the fast nonlinear blind decomposition (FNBD) method to enhance an impulsive signature [25], and Lei Wang et al. used ensemble local mean decomposition (ELMD) and fast kurtosis (FK) to extract an impulsive signal [26].

### 2.2. Convolutional Neural Network

CNN is extensively used to classify data. It is used primarily to extract features. Hence, it is easy to visualize models that can classify from one–dimensional to three–dimensional data including voices, animal images, and brain MRIs [27,28,29]. Both 1–D CNN and 2–D CNN are typically used in RFD, and their types are selected according to the dimension of the data [9,10,11,13,14,15,16,17,18,23]. The 1–D CNN and 2–D CNN mentioned in the abovementioned studies in Section 2.1 were used for 1–D data or 2–D data, respectively. 

### 2.3. Multi-Output Classification

There are four types of classification: binary classification, multi–class classification, multi–label classification, and multi–output classification [30]. Binary classification is used for targets with two classes and one label. Multi–class classification is used for targets with multiple classes (greater than two) and one label. Multi–label classification is used for targets with two classes and multiple labels. Multi–output classification is used for targets with multiple classes and labels. Dineva et al. proposed a multi–label classification–based model for the detection of multiple faults in rotating electrical machines [31]. Multi–class classification is used for single fault detection, whereas multi–label and multi–output classifications are used for compound fault detection. Multi–output classification is an effective technique because rotor systems usually encounter multiple faults.

## 3. Proposed Model

### 3.1. Data Acquisition

**Figure 1:**
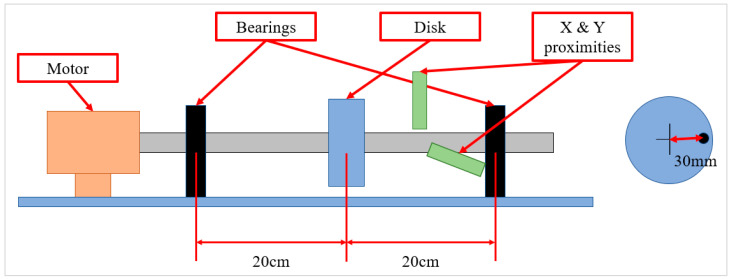
Schematic of the rotor kit.

**Figure 2:**
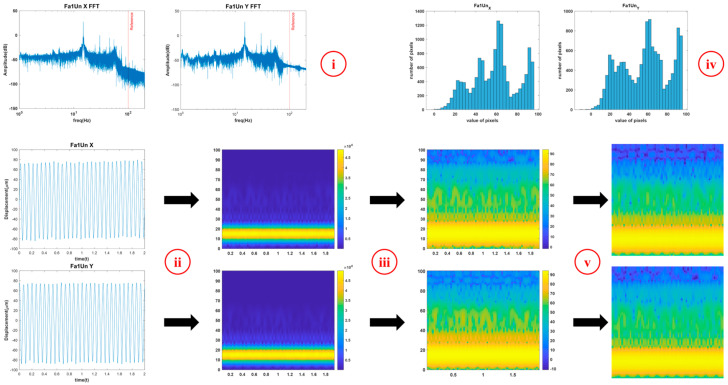
Data preprocessing using STFT. (**i**) Spectrums of the data obtained by FFT. Data under the frequency expressed with the red line are used. (**ii**) 2D spectrograms of the data obtained by STFT. (**iii**) Spectrograms converted to dB scale. (**iv**) Histograms used to determine the range of amplitude of spectrograms notable. (**v**) Preprocessed data by removing axes, labels, and color bar and resizing.

The transverse vibration data of the rotor kit were acquired from two perpendicular proximities, which measured X and Y displacements of the rotor shaft. The rotor shaft was supported by two ball bearings, as illustrated in Figure 1. Figure 2 shows the overall process of data preprocessing. The process consisted of the following steps:

1. Obtaining FFTs from raw data and determining the frequency limit that is unneglectable.
2. Obtaining STFTs from the raw data for the range determined at Step 1.
3. Converting the units of the values of STFTs to decibels (dB) to clarify the characteristics at high frequency range.
4. Determining the appropriate range of values to scale the STFTs from histograms.
5. Scaling using the range obtained from Step 4 and normalizing the size.

Only data below 100 Hz frequency were used, and the normalized size of the STFT images was 175 × 175. The overlap, window function, and sampling frequency used in STFT are 80%, Hanning window, and 18,000 Hz, respectively. STFT is a sequence of Fourier transforms of windowed signals as mentioned in Section 2, and the STFT pairs are obtained by:

(1) $$X_{STFT}\left[m,n\right]=\sum_{k=0}^{L-1}x\left(k\right)w\left(k-m\right)e^{-\frac{j2\pi nk}{L}},$$

(2) $$x\left(k\right)=\sum_{m}\sum_{n}X_{STFT}\left(m,n\right)w\left(k-m\right)e^{\frac{j2\pi nk}{L}},$$

where *x*(*k*) denotes a signal and *w*(*k*) denotes a window function. Herein, (1) is for obtaining the STFT and (2) is used for reconstructing.

Four classes, named normal, unbalance, one bearing fault, and two bearing faults, were studied. Unbalance and bearing faults are representative faults in rotor systems [5]. Unbalance mass influences the vibration of rotor systems, and this effect can be mathematically expressed as:

(3) $$y\left(t\right)=Ysin\left(\omega_{r}t-\theta \right),$$

(4) $$r=\frac{\omega_{r}}{\omega_{n}},$$

(5) $$Y=\frac{me}{M}\frac{r^{2}}{\sqrt{{\left(1-r^{2}\right)}^{2}+{\left(2\zeta r\right)}^{2}}},$$

(6) $$\theta =\tan^{-1}\frac{2\zeta r}{1-r^{2}},$$

where x is the displacement; X is the amplitude; M is the total mass of the system, including unbalanced mass; m is the unbalance mass; e is the distance of the unbalance mass; r is the ratio of the angular frequency ($\omega_{r}$) to natural frequency ($\omega_{n}$); ζ is the damping ratio; and θ is the phase of x. Note that me in (5) is crucial because the amplitude *Y* is proportional to me.

### 3.2. CNN–Based Model

**Figure 3:**
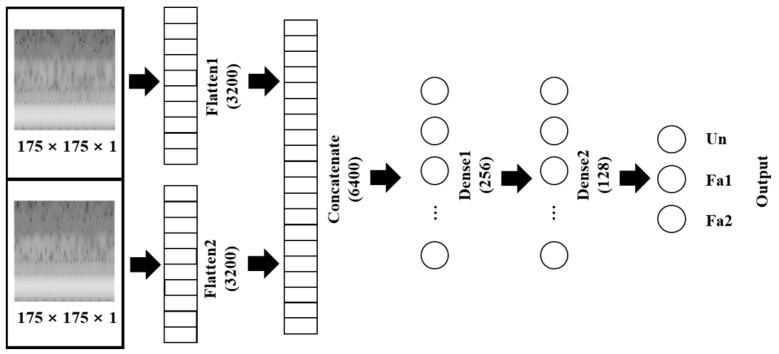
MLP–based model.

**Figure 4:**
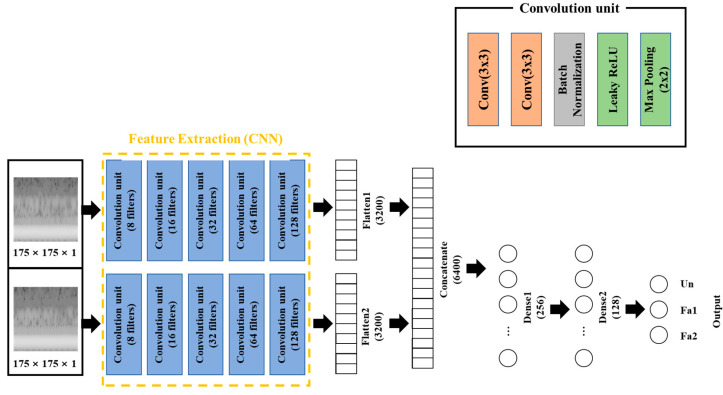
CNN–based model (proposed model). Convolution unit (blue) contains 5 steps consisting of convolution layer, batch normalization, activation function, and pooling layer. The number of filters written in the convolution unit means number of filters of the two convolution layers in the unit.

The conventional multi–class classification model and the proposed multi–output classification model are shown in Figure 3 and Figure 4. Figure 3 shows the MLP–based model. This model has the same structure as the CNN–based model except for the CNN part. The CNN layers had zero padding to prevent the loss of boundary information and had He initialization to improve the learning capability of the model. The abbreviation “Conv” in the convolution unit denotes “convolution layer”, which extracted features of input data by the operation expressed as:

(7) $$X_{o}=\varphi \left(WX_{i}+b\right),$$

where $X_{i}$ and $X_{o}$ denote the input and output of the layer, respectively; W denotes the weight array; b denotes the bias; and φ denotes the activation function applied on the summation. The batch normalization layer was used to prevent overfitting because it severely affected the test accuracy of the model. The *Leaky* rectified linear unit (*ReLU*) function was selected as the activation function of the model except for the output layers. *Softmax* was used to compare the results of the proposed model, whereas the *Sigmoid* function was used for the proposed model in the output layer.

The three activation functions are expressed as:

(8) $$Leaky\;ReLU=\left\{\begin{array}{cc} x, & x\geq 0\\ ax, & x<0 \end{array}\right.,$$

(9) $$Softmax=\frac{e^{x_{j}}}{\sum_{j}^{X}e^{x_{j}}}$$

(10) $$Sigmoid=\frac{1}{1+e^{-x}}$$

where *X* represents the number of classes. *Softmax* and *Sigmoid* are the representative activation functions in DL. *Softmax* is optimum for multi–class classification, whereas *Sigmoid* is optimum for multi–label classification. The multi–output classification had multi–labels. Hence, the proposed model used *Sigmoid* as the activation function of the output layer. *Leaky ReLU* is a modified form of the *ReLU* function, which is used to resolve the dead *ReLU* problem. The slope was determined in the negative region ‘a’ as 0.3. Loss function is an important component that affects backpropagation. In general, binary cross entropy (*BCE*) is used after a *Sigmoid* output, whereas categorical cross entropy (*CCE*) is used after a Softmax output. *BCE* and *CCE* can be expressed as:

(11) $$BCE=-\frac{1}{N}\sum_{i=0}^{n}t_{i}\cdot \log\left(y_{i}\right)+\left(1-t_{i}\right)\cdot \log(1-y_{i})$$

(12) $$CCE=-\frac{1}{N}\sum_{j=0}^{N}\sum_{i=0}^{C}t_{ij}\log\left(y_{ij}\right)$$

where *t* denotes the target label; *y* denotes the predicted probability; *N* denotes the number of samples; and C denotes the number of classes. *BCE* was the loss function of the proposed model because multi–output classification is a case of multi–label classification, whereas *CCE* was the loss function of the *Softmax* model for comparison. Adam optimizer, which combines the advantages of RMSProp optimizer and momentum optimizer, was used in this model.

**Figure 5:**
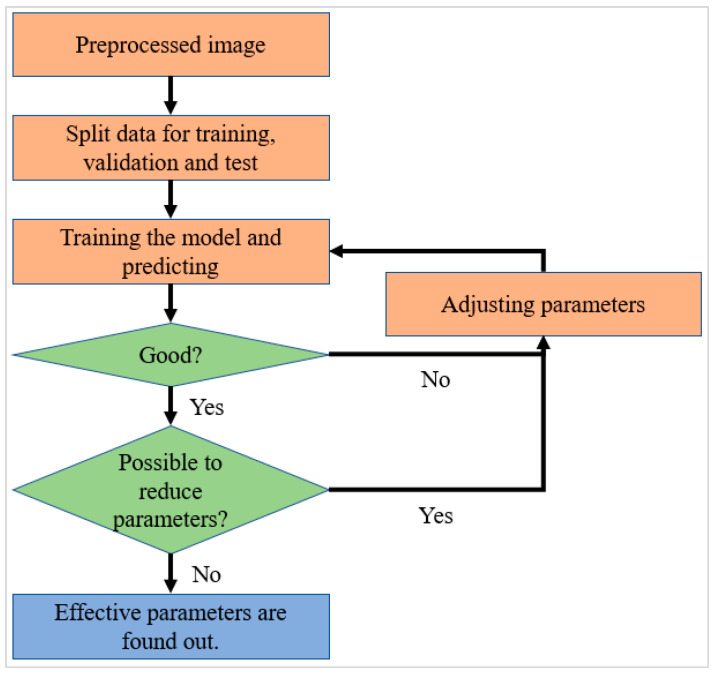
Flow chart for adjusting parameters.

Figure 5 shows the repetitive process as a flow chart, which was used for adjusting the parameters. The adjusted parameters included learning rate, epochs, and number of nodes of the layers after the concatenated layer. The learning rate is proportional to the weight change for update; thus, significantly high values of epoch can cause unnecessary learning and overfitting. The number of nodes affects the prediction and the number of parameters that require updates. Therefore, an appropriate learning rate was obtained in the range of 0.001–0.000001, and the repetitive process was 0.00001. The appropriate number of epochs was 50 and the appropriate number of nodes were 256 for the first layer and 128 for the second layer, as depicted in Figure 3 and Figure 4.

## 4. Experiments

### 4.1. Testbed and Properties

**Figure 6:**
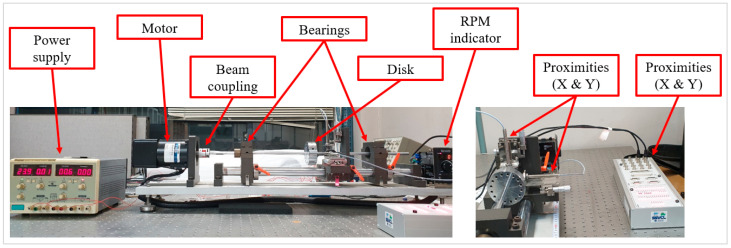
Rotor kit testbed.

**Figure 7:**
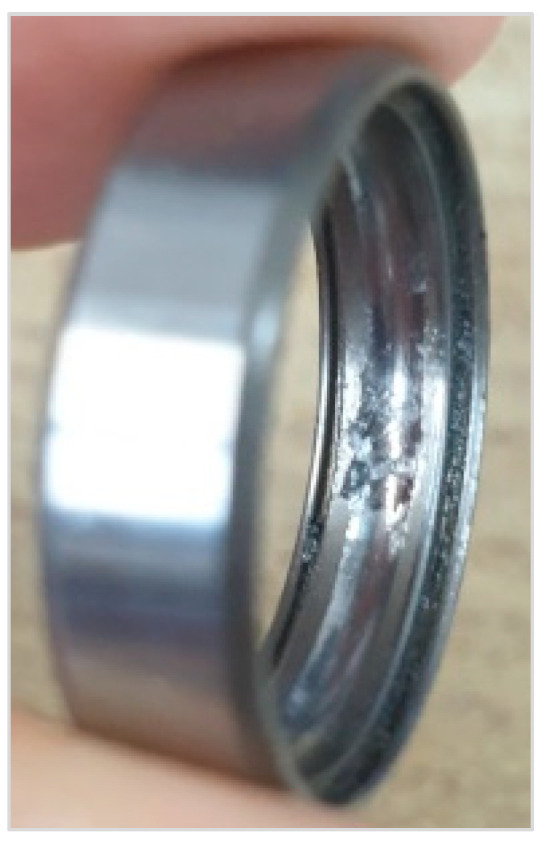
Wear on the outer race of the bearings.

The data were acquired from the rotor kit, as shown in Figure 6. Bently Nevada 3300 XL NSv Proximitors ^®^ Sensors were installed along the X and Y directions, and NSK 6800 VV deep groove ball bearings were inserted into the supporting parts. The raw data were obtained through dSpace RTI (real–time interface) 1104 in conjunction with MATLAB/Simulink. The main properties were the mass of the disk, distance between the disk and bearings, diameter of the shaft, and the unbalance for one unbalance bolt. An unbalanced bolt had a mass of 1.25 g, and the distance between the center of the shaft and unbalanced bolt was 30 mm. Hence, the value me in (5) was increased to 37.5g·mm per an additional unbalance bolt. The experiment was conducted under a fixed rotating speed at 900 rpm. Table 1 lists the main properties of the rotor kit testbed. The bearing fault type used was wear on the outer race of the bearings as shown in Figure 7.

### 4.2. Data Acquisition, Splitting and Labeling

**Figure 8:**
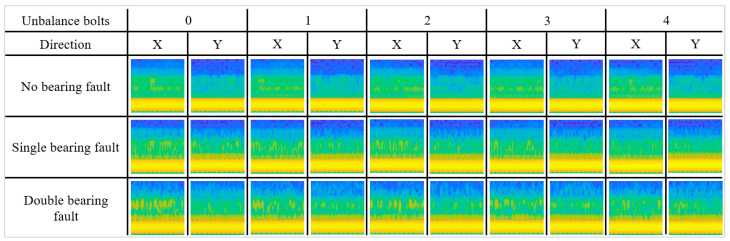
2D spectrograms of all types of faults.

The data for each case were acquired for 10 min at a sampling frequency of 18,000/s. The data of the four cases without fault (No) or single fault label (Un4, Fa1, Fa2) were used for training. The remaining 11 cases, which had a compound fault or ambiguous fault, were used for the test. Subsequently, the image data were obtained by determining the STFT of the raw data and Figure 8 shows the processed data. The preprocessed data were split into three sets: training, validation, and test dataset at a ratio of 70:15:15.

A total of 1800 images for each case were generated using the 2–second–long signal overlapping with 5/6 of the previous step. Hence, the size of the training set was 1260, whereas the size of the validation and test sets was 270. A summary of these cases is provided in Table 2. Herein, Fault1 implies a single bearing fault and Fault2 implies a double bearing fault. Numbers following the letters “Un” represent the number of the unbalanced bolts in the range of 1–4, with an unbalance in the range of 37.5–150 g·mm. Un1–Un3 represent ambiguous fault types. A vector indicating probabilities with a form of [Un, Fa1, Fa2] was used as the labels. In contrast to general labeling methods, this study did not consider the normal state as a label because it can be expressed as a label [0, 0, 0].

## 5. Experiments

**Figure 9:**
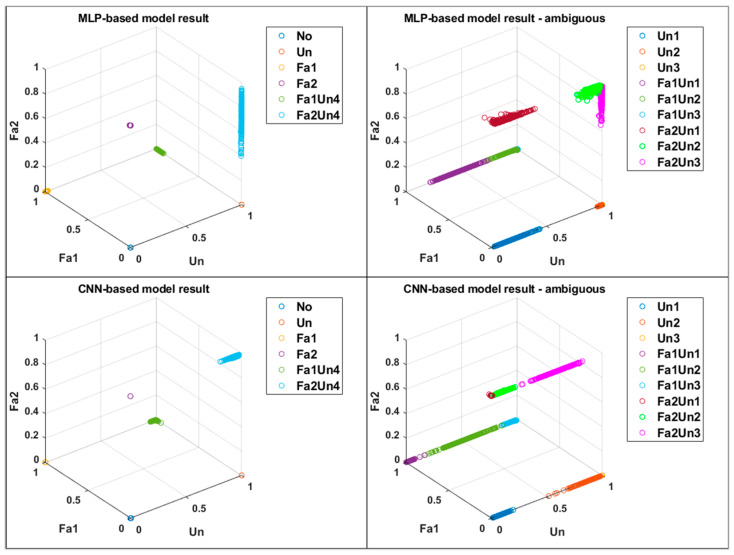
3D scattered probability plot of the MLP–based and CNN–based models.

**Figure 10:**
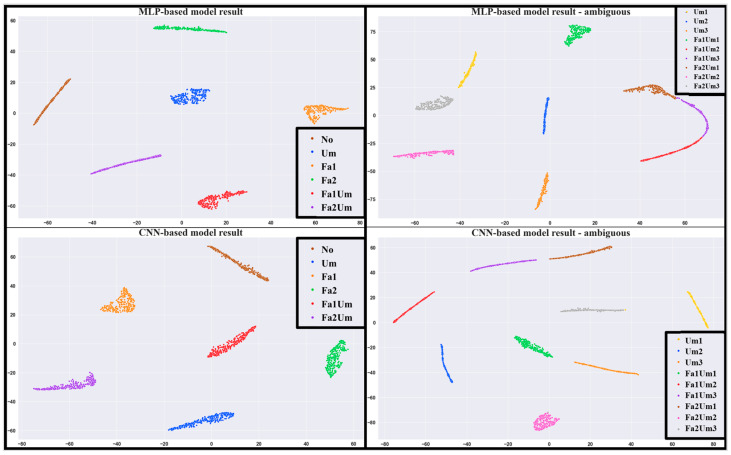
t–SNE results of the (**left**) MLP–based model and (**right**) CNN–based model.

Three types of faults were observed in this study. Therefore, the prediction was visualized using a three–dimensional scatter plot, as shown in Figure 9, wherein each axis indicates the probabilities of Unbalance, Fault1, and Fault2. The MLP–based model exhibited poor prediction of the Fa2 type, whereas the CNN–based model exhibited an optimum result. These images demonstrate the following two major advantages of the proposed models: First, the proposed models independently predicted untrained compound faults and these faults did not affect the probabilities of the remaining types of faults. Second, the proposed models were robust to the change in the degree of unbalance even though the models were not trained for ambiguous faults. The number of parameters was crucial because a large number of parameters represents a complex model. Hence, it requires a long time for training and prediction. Table 3 compares the number of parameters in the MLP–based and CNN–based models.

t–distributed stochastic neighbor embedding (t–SNE) is a popular clustering method used to visualize high–dimensional data by reducing the dimensionality [28]. Figure 10 shows the clustered result using t–SNE.

**Figure 11:**
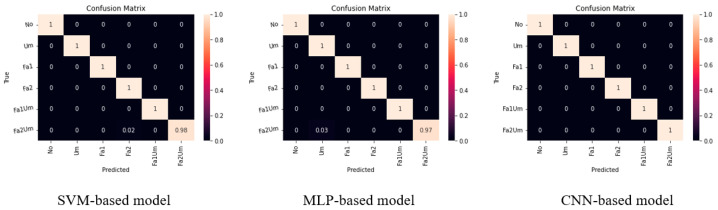
Confusion matrices of SVM–based, MLP–based and CNN–based models.

Additionally, confusion matrix is another method used to evaluate the performance of models. The three matrices in Figure 11 were obtained with the simplest condition that it is correct when the probability was greater than 0.5 because the models used the *Sigmoid* function as the activation function of the output nodes. The matrices demonstrated that the CNN–based model had the best performance, whereas the MLP–based and SVM–based models performed slightly worse than that of the CNN–based model.

Table 4 lists the mean and variance values of the result of the CNN–based proposed model for ambiguous faults. It can be inferred from the shape of the *Sigmoid* function that a higher value of variance was obtained when the average value was closer to 0.5. The model performed worse for the probability of unbalance when Fault2 was included. Additionally, the model had a poor performance for the smallest unbalance data. It can be observed from Table 4, Figure 9 and Figure 10 that the CNN–based proposed model appropriately separated ambiguous faults.

Table 5 shows the F1 score of the SVM–, MLP–, and CNN–based models. F1 score is an evaluation index obtained from the predicted value and true value of a model [29]. F1 score also achieved a nearly perfect result, as the confusion matrix showed.

## 6. Conclusions

A model with only single fault data was trained and used to diagnose compound faults. The two main characteristics required in the model were the accuracy for compound faults and independence of each probability. Multiple studies have proposed AI models with exceptional performance for single faults. Therefore, an efficient model for complex faults was proposed. There are multiple possible cases of faults in rotor systems. Hence, the data acquisition of compound faults was performed considering the number of all cases. The proposed model demonstrated accurate classification for single and compound faults with the same level of the trained data and classification ability for ambiguous faults. Therefore, this model, which performed multi–output classification, can be used with appropriate thresholds in industries.

## Figures and Tables

**Table 1:** Properties of the rotor kit testbed.

Disk Mass [g]	Distance [cm]	Shaft Diameter [mm]	Unbalance for One Bolt [g · mm]	Rotating Speed [RPM]
770	20	10	37.5	900

**Table 2:** Data categories.

	Case ID	Fault Type	Label	Unbalance [g · mm]
Trained	No	Normal	[0, 0, 0]	0
	Un4	Unbalance	[1, 0, 0]	150
	Fa1	Fault1	[0, 1, 0]	0
	Fa2	Fault2	[0, 0, 1]	0
Untrained	Un1	Unbalance	[1, 0, 0]	37.5
	Un2	Unbalance	[1, 0, 0]	75
	Un3	Unbalance	[1, 0, 0]	112.5
	Fa1Un1	Fault1 + Unbalance	[1, 1, 0]	37.5
	Fa1Un2	Fault1 + Unbalance	[1, 1, 0]	75
	Fa1Un3	Fault1 + Unbalance	[1, 1, 0]	112.5
	Fa1Un4	Fault1 + Unbalance	[1, 1, 0]	150
	Fa2Un1	Fault2 + Unbalance	[1, 0, 1]	37.5
	Fa2Un2	Fault2 + Unbalance	[1, 0, 1]	75
	Fa2Un3	Fault2 + Unbalance	[1, 0, 1]	112.5
	Fa2Un4	Fault2 + Unbalance	[1, 0, 1]	150

**Table 3:** Number of parameters in MLP and CNN based models.

Model Types	Number of Parameters
MLP	64.08 M
CNN	2.3 M

**Table 4:** Mean and variance of the result from CNN–based model in Figure 9.

Fault Type	Mean	Variance
Um1	0.0658	0.0012
Um2	0.8735	0.0049
Um3	0.9986	0.0000
Fa1Un1	0.0266	0.0003
Fa1Un2	0.5320	0.0174
Fa1Un3	0.9567	0.0004
Fa2Un1	0.0036	0.0000
Fa2Un2	0.0870	0.0013
Fa2Un3	0.5923	0.0106

**Table 5:** F1 score of MLP– and CNN–based models.

Model Types	F1 Score
SVM	99.6 %
MLP	99.5 %
CNN	100 %

## Data Availability

Not applicable.
